# Preliminary results of a feasibility study of the use of information technology for identification of suspected colorectal cancer in primary care: the CREDIBLE study

**DOI:** 10.1038/bjc.2015.45

**Published:** 2015-03-03

**Authors:** E Kidney, L Berkman, A Macherianakis, D Morton, G Dowswell, W Hamilton, R Ryan, H Awbery, S Greenfield, T Marshall

**Affiliations:** 1 Primary Care Clinical Sciences, School of Health and Population Sciences, University of Birmingham, Birmingham, West Midlands B15 2TT, UK; 2 Department of Surgery, School of Cancer Sciences, Queen Elizabeth Hospital Birmingham (old), University of Birmingham, Room 29, 4th Floor, Edgbaston, Birmingham B15 2TT, UK; 3 College House, University of Exeter Medical School, St Luke's Campus, Exeter EX1 2LU, UK

**Keywords:** colorectal cancer, primary care, diagnosis, electronic medical records

## Abstract

**Background:**

We report the findings of a feasibility study using information technology to search electronic primary care records and to identify patients with possible colorectal cancer.

**Methods:**

An algorithm to flag up patients meeting National Institute for Health and Care Excellence (NICE) urgent referral criteria for suspected colorectal cancer was developed and incorporated into clinical audit software. This periodically flagged up such patients aged 60 to 79 years. General practitioners (GPs) reviewed flagged-up patients and decided on further clinical management. We report the numbers of patients identified and the numbers that GPs judged to need further review, investigations or referral to secondary care and the final diagnoses.

**Results:**

Between January 2012 and March 2014, 19 580 records of patients aged 60 to 79 years were searched in 20 UK general practices, flagging up 809 patients who met urgent referral criteria. The majority of the patients had microcytic anaemia (236 (29%)) or rectal bleeding (205 (25%)). A total of 274 (34%) patients needed further clinical review of their records; 199 (73%) of these were invited for GP consultation, and 116 attended, of whom 42 were referred to secondary care. Colon cancer was diagnosed in 10 out of 809 (1.2%) flagged-up patients and polyps in a further 28 out of 809 (3.5%).

**Conclusions:**

It is technically possible to identify patients with colorectal cancer by searching electronic patient records.

Colorectal cancer is the UK's fourth most common cancer (Cancer Research UK, 2013). Poorer survival rates have been noted in Denmark and the UK than in other countries, partly owing to treatment at a later stage (Gatta *et al*, 2003; Iversen, 2012; Hamilton *et al*, 2013; Maringe *et al*, 2013) There are a number of intervals in the diagnostic process, each contributing to the overall period of time between symptom onset and treatment. There are intervals between first experience of symptoms and consultation; between consultation and having symptoms meeting referral criteria; between meeting referral criteria and being referred for diagnostic investigations; and an interval between diagnosis and treatment (Weller *et al*, 2012). There is evidence that a shorter interval between presenting with symptoms in primary care and diagnosis is associated with improved prognosis (Tørring *et al*, 2011; Neal *et al*, 2015). Despite modest declines in the interval between meeting referral criteria and diagnosis after the introduction of referral guidelines in 2005, one-quarter of patients meeting National Institute for Health and Care Excellence (NICE) 2005 referral criteria experience delays of over 5 months (NICE, 2005; Neal *et al*, 2014).

Prediction models show some promise in assisting earlier identification of colorectal cancer. Recent years saw the development and validation of the CAPER scoring system to identify patients with suspected colorectal cancer (Hamilton, 2009; Marshall *et al*, 2011). Since then, a prediction model to determine cumulative incidence of colorectal cancer from information available in electronic primary care records has been developed and validated (Collins and Altman, 2012; Hippisley-Cox and Coupland, 2012). A number of strategies making use of such prediction models have been proposed to reduce delays between symptomatic presentation and diagnosis of colorectal cancer. Desk-based risk assessment tools have been provided for use during consultation (Hamilton *et al*, 2013). These tools remind GPs that a patient's symptoms or clinical features indicate an increased cancer risk. A related approach in primary health-care systems that use electronic primary care records is to use electronic prompts. These automatically flag up patients at the time of consultation whose symptoms or clinical features indicate a pre-specified risk of cancer. Both of these approaches assist GPs in identifying potential cancer symptoms in patients as they consult.

Another strategy is to actively search electronic primary care records for patients with symptoms or clinical features that indicate that they meet referral criteria for colorectal cancer or a pre-specified risk of cancer. This means that, once identified, patients can be invited to attend the surgery for further investigation. This approach has practical advantages over reminders or tools used during consultation, as it identifies patients who may have been missed previously, either because no action was considered necessary at the time of consultation or because the relevant clinical information was entered after the consultation had ended, meaning that an electronic prompt would be activated too late. However, there are challenges to implementing such a strategy. Information technology must be developed to identify the correct patients, practices must review the records of identified patients and appropriate clinical action must be taken. This paper reports the findings of CREDIBLE (ColoRectal Early Diagnosis: an Information Based Local Evaluation), a feasibility study to investigate the use of information technology to search electronic primary care records and to identify patients who meet NICE 2005 urgent referral criteria for suspected colorectal cancer (NICE, 2005).

The study had several objectives. The first objective was to produce and deploy a software algorithm in practice systems that would identify patients meeting NICE 2005 urgent referral criteria for suspected colorectal cancer (NICE, 2005). Process objectives were to determine the proportion of patients identified as meeting NICE (2005) urgent referral criteria; the proportion of identified patients subsequently referred for investigation and to describe the main reasons why patients might not be referred; the final diagnoses ascribed to identified patients; and the relationship of referral to participation in the bowel cancer screening programme. A separate paper will report the results of interviews investigating the acceptability of the system to participants and to GPs.

## Materials and methods

General practices were invited by letter and e-mail to take part in the study and promotion by local Primary Care Trust (PCT) nurse screening teams and the cancer leads, an article in the Primary Care Research Network newsletter and a GP education session on colorectal cancer. Participating general practices were in the urban West Midlands, UK, and had access to software that could be adapted for the study. The target population was patients aged 60 to 79 years without a previous diagnosis of colorectal cancer. Younger patients were excluded because of their low incidence of colorectal cancer, and older patients were excluded because co-morbidities and contraindications to further investigation were likely to be common.

Patients aged ⩾60 years meet NICE 2005 urgent referral criteria if they have any of the following clinical features: diarrhoea or looser stools for ⩾6 weeks; rectal bleeding for ⩾6 weeks; haemoglobin ⩽11 g dl^−1^ in men or ⩽10 g dl^−1^ in women, accompanied by iron deficiency (NICE, 2005).

In collaboration with a medical software company, MSDi (Merck Sharp and Dohme, Hertfordshire, UK), we adapted Clinical Manager software to enable searches on electronic primary care records. Searches identified patients without a previous diagnosis of colorectal cancer whose records indicated that they met referral criteria up to 2 years before the date of the search. The new software query was incorporated into Clinical Manager software as part of a routine upgrade and was activated in practices agreeing to participate in the study. This software query was run as an add-on to existing clinical records systems.

Translation of urgent referral criteria into clinical coding followed a previously described method and was partly informed by a previous analysis of predictors of colorectal cancer (Marshall *et al*, 2011). Six weeks of looser stools was indicated by two clinical codes for diarrhoea or new prescriptions for anti-diarrhoeal drugs separated by at least 35 days but fewer than 119 days, or by a single clinical code for change in bowel habit. Rectal bleeding for 6 weeks was indicated by a single clinical code for rectal bleeding. Iron deficiency anaemia was indicated by appropriate haemoglobin values accompanied by mean red cell volumes ⩽80 fl. In addition, patients were considered eligible for urgent referral if they had either a positive faecal occult blood test (FOBt), an abdominal mass or an abnormal rectal examination suggestive of a rectal abnormality.

It was intended to run the software weekly in participating general practices and to produce a list of patients flagged up as meeting urgent referral criteria. As GP records do not code reasons for referral, it was not possible to exclude patients who had already undergone investigation for suspected cancer without checking individual medical records. Patients who were flagged up and reviewed once were not reviewed a second time if they were flagged up again.

GPs were briefed by the researcher and a study pack was left for reference. We followed this up with individual contacts, practice visits, emails and newsletters throughout the study. Participating practices agreed to review the list of flagged-up patients either using their own staff or supported by a nurse seconded to the practice and to determine whether patients had already been referred for investigation for possible colorectal cancer; had another diagnosis explaining their clinical features; investigation was contraindicated or had been declined; and they needed further review by a clinician.

Practices provided anonymised data including patients' basic demographic details to determine number of eligible patients and clinical information relevant to the project, including reasons for not investigating patients who were flagged up. Problems were logged.

### Analysis

We present descriptive statistics: the incidence of flagged-up patients, their symptoms, proportions reviewed in primary care, proportions investigated and diagnoses. We also describe the principal reasons given for not investigating flagged-up patients. Comprehensive analysis of variations between practices of incidence of flagged-up patients, symptom patterns and diagnoses of flagged-up patients will be the subject of a future paper.

We planned to run the searches in 20 practices, covering a total registered population of ∼120 000, including 20 000 aged 60 to 79 years. It was anticipated that this could identify ∼1000 patients aged 60 to 79 years who were eligible for urgent referral.

Ethical approval was granted by the NHS National Research Ethics Service (Reference 11/WM/0404).

## Results

### Practices and population searched

Between January 2012 and March 2014, records were searched electronically in 20 general practices in Sandwell and Dudley. Practices were in relatively deprived areas and had a mean registered population of 5522 (range 2085 to 13 996). Of the total registered population of 110 435 patients, 19 580 patients were aged 60 to 79 years. Searches were run monthly because data were uploaded monthly from practice records to MSDi Clinical Manager. Technical delays meant that in some practices searches took place less frequently. Several practices changed their clinical records systems, and as a result new data extraction procedures needed to be developed. (For a summary of the implementation process and problems encountered, see Supplementary Online Material 1)

### Number of patients meeting referral criteria

Of 19 580 eligible patients, 809 (4.1%) were identified as meeting urgent referral criteria: 416 (51.4%) were female; their average age was 69 years. Mean age and gender distribution were similar across all 20 practices.

The first search conducted identified a backlog of prevalent cases who had met referral criteria over the previous 2 years. These first searches covered ∼39 160 person-years of follow-up, equivalent to a mean incidence of 11.3 flagged-up patients per 1000 person-years of follow-up.

Subsequent searches were undertaken for a median of 17 months, each search identifying smaller numbers of newly incident cases. In the 25 776 person-years of follow-up covered in subsequent searches, the mean incidence of flagged-up patients was 12.4 per 1000 person-years.

Microcytic anaemia, rectal bleeding and persistent diarrhoea were the commonest clinical features resulting in patients being flagged up. The pattern of clinical features was similar in prevalent and newly incident cases (Table 1).

### Findings after clinical review of patient records

The records of the 809 flagged-up patients were reviewed by a clinician in each practice (in 19 a nurse; in 1 a GP) and categorised into two groups: (1) for further review of their records by the GP; and (2) not for further review. Those not for further review were sub-categorised as: either having previously been referred for investigation; or on the basis of clinical judgment, having other reasons not to have further review. In all, 274 (34%) of the flagged-up patients were judged by the clinician as needing further review; a small number of these had previously been referred or had other possible explanations for their symptoms. Of those not for further review, 428 (53%) had previously been referred (Table 2); 107 (13%) had other reasons not to investigate further, such as other cancers, peptic ulcers, advanced renal disease, chronic bowel conditions, iatrogenic or medication side effects, infections and extensive haemorrhoids, frail patients and those receiving palliative care. Three patients were prescribed codeine for pain relief unrelated to bowel disease.

The most frequent clinical feature judged to require further review was microcytic anaemia (112 cases) followed by persistent diarrhoea (70) and rectal bleeding (52); (Table 3). There were marked differences in the proportion of clinical features for which patients had been referred for investigation: 89% of those who were FOBt positive; 68% of those with rectal bleeding; 67% of those with change in bowel habit; 35% of those with persistent diarrhoea; 40% of those with microcytic anaemia; 38% of those with an abdominal mass (*χ*^2^ *P*<0.001). After excluding the 428 patients whose previous referral precluded the need for further review, 71.9% (274 out of 381) of the remaining patients needed further review. This proportion was very similar across all clinical features (Table 3). Reasons for GPs deciding not to invite patients included the patient having undergone lower gastrointestinal investigations some years previously, a negative FOBt, the symptoms having been present for many years and therefore cancer considered to be less likely, symptoms having resolved, not considering that diarrhoea or anaemia might indicate a risk of bowel cancer and patients moving or dying since the search was undertaken.

A slightly lower proportion (30.5% (152 out of 498)) of patients flagged up at first search (prevalent cases) needed further review than the proportion (39.2% (122 out of 311)) flagged up at subsequent searches (incident cases; *χ*^2^ *P*=0.002). However, in both prevalent and incident cases, the pattern of previous referral and decisions to undertake further clinical review in relation to clinical features was similar.

### Further investigations

Following GPs' inspections of the records of 274 patients flagged up as being in need of further review, it was decided to invite 72.6% (199 out of 274) for a consultation, and not to invite 22.6% (62 out of 274). In addition, four had died or moved away from the area/practice, and three were considered to have more pressing co-morbidities. We were unable to obtain information for six patients. When grouped by their presenting symptoms, there was only minor variation in the proportion of patients that GPs decided to invite for consultation. However, one practice invited no patients.

Of the invited patients, 58.3% (116 out of 199) attended and were seen by their GP; the remainder declined or did not respond to the invitation. A similar proportion of first search (prevalent) and subsequent search (incident) cases attended appointments with their GPs. Of these, 36.2% (42 out of 116) were referred for further investigation in secondary care: 33 to colorectal surgeons, 8 to gastroenterology and 1 to haematology. Of the remaining patients, 36.7% (31 out of 116) had further investigations in primary care (FOBt, full blood counts or both) and 37.1% (43 out of 116) had no further investigations (Table 4). Similar proportions of first search (prevalent) and subsequent search (incident) cases were referred to secondary care. There was no evidence of variation in referrals or in primary care investigations by presenting symptoms. Analysis of variations in primary care investigations using funnel plots indicated that two general practices undertook FOBt in most patients and one undertook full blood counts in most patients and that these variations were not consistent with chance (See Supplementary Online Material Figures 1 and 2).

Of the 42 referrals, 29 patients underwent further investigations, 7 attended but did not undergo further investigations (2 because they declined investigation, and 5 because symptoms had resolved), 1 attended haematology only and 5 did not attend (1 patient denied symptoms, 2 patients had a history of non-attending and 2 became either frail or confused).

### Cancers and other diagnoses

Of the 809 patients flagged up, 1.2% (10 out of 809) were subsequently diagnosed with colorectal cancer (all were colon cancers): 1.0% (5 out of 498) of those flagged up in first searches and 1.6% (5 out of 311) of subsequent searches (Fisher's exact test, two-tailed *P*=0.520). This is equivalent to incidence rates of 12.8 and 19.4 per 100 000 person-years in first and subsequent searches, respectively. Patients who were not reviewed or investigated further could present again to their GPs and be investigated and diagnosed independently of this study. Colon cancers were diagnosed in 1.8% (5 out of 274) patients whose records were selected for GP review and in 0.9% (5 out of 535) not selected for GP review (all because they had previously been referred for investigation; Fisher's exact test, two-tailed *P*=0.320). Six colon cancers were diagnosed in patients flagged up because of iron deficiency anaemia and four because of rectal bleeding.

Twenty-eight patients were diagnosed with colorectal polyps, giving a total of 38 with either colorectal polyp or cancer. For the combined diagnosis of colorectal polyp or cancer, this is equivalent to incidence rates of 43.4 and 81.5 per 100 000 person-years in the first and subsequent searches. Colorectal cancer or polyp was diagnosed in 4.7% (13 out of 274) of patients whose records were selected for GP review and in 4.7% (25 out of 535) of patients not selected for GP review. (*χ*^2^ *P*=0.964) Of these not for GP review, 23 patients had already been referred, 1 had subsequent new symptoms and 1 had symptoms previously ascribed to medication side effects. Of those judged to need GP review, colorectal cancer or polyp was diagnosed in two patients who were not invited for a GP consultation, in one patient who did not attend the GP consultation, in three patients who were not referred to secondary care and in seven of those referred to secondary care (17% of those referred).

Of those diagnosed with either cancer or polyp, 45% (17 out of 38) were flagged up because of rectal bleeding, 29% (11 out of 38) because of iron deficiency anaemia, 11% (4 out of 38) because they screened FOBt positive, 9% (3 out of 38) because of change in bowel habit and 9% (3 out of 38) because of persistent diarrhoea.

In total, 15.1% (122 out of 809) of flagged-up patients were given at least 1 new diagnosis following CREDIBLE. This included 17 non-colorectal cancers, 33 diverticulosis or diverticulitis, 31 anal fissures or haemorrhoids, 6 upper gastrointestinal diagnoses (peptic ulcer or gastritis), 5 colitis or proctitis, 4 bile salts malabsorption or irritable bowel syndrome, 2 colonic telangactasia or angioma and 2 volvulus. There was no statistically significant difference in the yield of new diagnoses between flagged patients selected for GP review (12.4% (34/274)) and those not selected for review (16.4% (88/535) (*χ*^2^ *P*=0.129)).

### Time to investigation and diagnosis

Partial data were available on the time intervals between symptoms, patients being flagged up, review of records, GP consultation and definitive investigation in secondary care.

For patients flagged up at first search (prevalent cases), the median intervals (interquartile ranges and numbers) were as follows: from search to review of patient records, 0 days (IQR: 0 to 8; *n*=491); from search to being seen by the GP, 73 days (IQR 61 to 109, *n*=44); and from search to secondary care investigation, 161 days (IQR: 79 to 243; *n*=20). Median interval from symptoms to being flagged up was 362 days (IQR: 188 to 568; *n*=430).

For patients flagged up in subsequent searches (incident cases), the median intervals were as follows: from search to review of patient records, 0 days (IQR: 0 to 0; *n*=308); from search to being seen by GP, 45 days (IQR: 21 to 179; *n*=34); and from search to secondary care investigation, 71 days (IQR: 40 to 120; *n*=16). Median interval from symptoms and being flagged up was 53 days (IQR: 37 to 90; *n*=297).

A number of flagged-up patients had previous symptoms that had already been investigated, 282 with symptoms and 80 as a result of positive FOBt screening tests from the bowel cancer screening programme. In comparison with the intervals observed in patients flagged up as part of this study, median intervals from symptoms to secondary care investigation were 18 days (IQR: 13 to 24 days) for patients identified through FOBt screening and 40 days (IQR: 15 to 87 days) for patients with symptoms.

## Discussion

The software algorithm successfully flagged up patients meeting referral criteria for suspected bowel cancer. Two-thirds of these flagged-up patients had not previously been referred, notably those with anaemia or persistent diarrhoea. Thirty-four per cent of flagged-up patients were judged to require GP review; 23% were invited for GP consultation; 14% attended a GP consultation; and 5% were referred for investigation in secondary care. Searches identified 10 colon cancers (1.2% of the patients flagged up): based on national incidence rates, we would expect 58 cases in the total population searched over this period of time. Searches also identified 28 colorectal polyps and a number of other significant diagnoses.

A future qualitative paper will examine the process involved in implementing our algorithm; however, implementation was not always smooth. There was evidence that general practices lacked capacity, skills and processes to deal with a list of patients meeting referral criteria for suspected bowel cancer. In some general practices, despite repeated reminders by research staff, the list of patients selected for GP review was not reviewed for many weeks or months. In one instance, a patient died of colorectal cancer several months after being flagged up but before their records had been reviewed. Some GPs expressed surprise that patients with iron deficiency anaemia but without gastrointestinal symptoms were eligible for investigation. GPs were unclear whether a negative FOBt or an improvement in iron deficiency anaemia after iron supplementation made referral unnecessary. There was no evidence that the one-third of flagged patients selected for GP review of their records were more likely to have colorectal cancer or polyps than the two-thirds who were not selected. Future implementation might consider selecting for further review all flagged-up patients whose recent symptoms have not been investigated. A substantial number of patients did not attend when invited for consultation. There was variation in the process of investigation and evidence that some practices used FOBt or full blood counts to obviate referral. It is arguable that patients flagged up warranted definitive investigation rather than FOBt or full blood counts, as these are insufficiently accurate tests to rule out cancer. Referrals were not always made to investigate the lower gastrointestinal tract, referrals were not always urgent (via the 2-week wait pathway), some patients did not attend their appointments and some were not investigated in secondary care because symptoms seemed to resolve.

There was also evidence of delay in investigation. Because data were uploaded monthly, there was typically up to 2 months between patients reporting incident symptoms and the software flagging them up. Review of flagged-up patients by a nurse was very prompt, but additional clinical research support was provided for this. A median of over 2 months elapsed between incident cases being flagged up and diagnostic investigations in secondary care. Those flagged up in this study were investigated less promptly than patients who had previously been investigated. Future implementation might include clear protocols for prompt referral for lower gastrointestinal investigation.

A longer period of follow-up might elicit further important diagnoses, particularly in those patients who were not investigated. Overall, the study demonstrated that although it is technically possible to identify patients with possible but uninvestigated colorectal cancer, the present configuration of general practice is ill suited to make use of this information. We observed a median of only 18 days between patient identification as part of bowel cancer screening programme and secondary care investigation. Referral and investigation are standard practice following a positive screening test, with clear pathways and protocols for investigation. For active flagging up of symptomatic patients to be effective, similar processes may need to be put in place.

The study made use of existing guidelines to identify patients for further investigation, but since 2014 it has become possible to undertake similar searches using the QCancer prediction model within EMIS-Web clinical records software (Hippisley-Cox and Coupland, 2013a, 2013b; Hippisley-Cox, 2014). Similar efforts to develop functionality have been shown with the Macmillan eCDS tool on the BMJ Informatica platform (Moffat *et al*, 2014). A more sophisticated prediction model, integrated with existing clinical records software, may improve discrimination characteristics, reduce the technical problems and shorten the interval between symptoms and flagging up patients. However, technical improvements do not address the problems of delay in investigating flagged-up patients or inconsistency in investigation and referral. Indeed, flagging up the risk of multiple cancers with different investigations and referral pathways raises additional challenges.

The number of cancers, polyps and other diagnoses emerging from the investigated patients in this study suggest that implementation of the algorithm is feasible. However, before recommending widespread implementation, we need further understanding of the resource implications, the effects on GP practices and on patients.

## Figures and Tables

**Table 1:** Clinical features of patients flagged up as meeting colorectal cancer referral criteria

**Clinical feature**	**Identified in first search (prevalent cases)**		**Identified in subsequent searches (incident cases)**		**Total**	
Low haemoglobin and microcytic anaemia	146	29%	90	29%	236	29%
Rectal bleeding	118	24%	87	28%	205	25%
Diarrhoea for ⩾6 weeks	106	21%	52	17%	158	20%
Change in bowel habit	65	13%	39	13%	104	13%
FOBt positive from bowel cancer screening *α*	54	11%	40	13%	94	12%
Abdominal or rectal mass *β*	5	1%	3	1%	8	1%
Symptoms not collected *γ*	4	1%		0%	4	0%
Total	498	100%	311	100%	809	100%

Abbreviation: FOB=faecal occult blood.

*α* includes one FOB result incorrectly recorded as positive. *β* no rectal masses were recorded. *γ* not recorded by nurse reviewing patient notes.

**Table 2:** Number of flagged-up patients identified as needing further review and reasons given why they might not need further review

**Clinician judgement about the need for further review**	**Identified in first search (prevalent cases)**		**Identified in subsequent searches (incident cases)**		**Total**	
**Clinician judges further review is needed**						
Previously referred^a^	10	2%	10	3%	20	2%
Other possible explanations for symptoms	5	1.0%	5	2%	10	1%
Contraindications to further investigation			1	0%	1	0.1%
Declined referral or did not attend			3	1%	3	0.4%
None of the above	137	28%	103	33%	240	30%
**Clinician judges further review is not needed**						
Previously referred^a^	266	53%	162	52%	428	53%
Other possible explanations for symptoms	47	9%	17	5%	64	8%
Contraindications to further investigation	14	3%	3	1%	17	2%
Declined referral or did not attend	4	0.8%	1	0%	5	0.6%
Moved	3	1%	1	0%	4	0.5%
Died	5	1%	4	1%	9	1%
Records missing or unavailable	7	1.4%	1	0%	8	1.0%
Total	498	100%	311	100%	809	100%

Abbreviation: IQR=interquartile range.

a Investigated a median of 194 days (IQR 63 to 415; *n*=344) previous to the search.

**Table 3:** Clinical features of flagged-up patients and decisions made about further clinical review

			**Not for further review**					
**Clinical features**	**For further review**		**Previously referred**		**Other reasons**		**Total**	
FOBt positive from bowel cancer screening	7	7%	84	89%	3	3%	94	100%
Change in bowel habit	27	26%	69	66%	8	8%	104	100%
Diarrhoea for ⩾6 weeks	70	44%	51	32%	37	23%	158	100%
Microcytic anaemia	112	47%	89	38%	35	15%	236	100%
Abdominal mass	3	38%	3	38%	2	25%	8	100%
Symptoms not collected	3	75%	1	25%		0%	4	100%
Rectal bleeding	52	25%	131	64%	22	11%	205	100%
Total	274	34%	428	53%	107	13%	809	100%

Abbreviation: FOBt=faecal occult blood test.

**Table 4:** Referrals and further investigations following GPs consultation

**Referrals**	***n***	**%**	**Further investigations**	***n***	**%**
Referred to colorectal surgeons	33	28	Colorectal surgery referral only	28	24
			Full blood count, then colorectal surgery referral	3	3
			Full blood count and faecal occult blood test, then colorectal surgery referral	2	2
Referred to gastroenterology or haematology	9	8	Gastroenterology	7	6
			Gastroenterology and haematology	1	1
			Haematology	1	1
Not referred: further investigations in primary care	31	27	Faecal occult blood test only	12	10
			Full blood count	11	9
			Full blood count and faecal occult blood test	7	6
			Full blood count and faecal occult blood test–follow-up declined by patient	1	1
Not referred: no specific investigations	43	37	Keep under review	3	3
			None–not applicable^a^	31	27
			Follow-up declined by patient	9	8
Total	116	100		116	100

Abbreviation: GPs=general practitioners.

a Includes one case in which the decision was taken after discussion with a colorectal surgeon and one case that had already been referred for upper, rather than lower, gastrointestinal tract investigation

## Notes

WH is a clinical advisor for the current revision of the NICE 2005 guidance, CG 27, on the topic of selection of patients for cancer investigation. His contribution to this article is in a personal capacity, and is not to be interpreted as representing the view of the Guideline Development Group, or of NICE itself.
