# Supplementary material for: Preliminary results of a feasibility study of the use of information technology for identification of suspected colorectal cancer in primary care: the CREDIBLE study
Source: Br J Cancer. 2015 Mar 3;112(Suppl 1):S70–6. doi: 10.1038/bjc.2015.45 (PMC4385979; doi:10.1038/bjc.2015.45)
Supplement: Supplementary Figure Legends [file bjc201545x3.doc]

Supplementary Online Material 1: Implementation process and examples of problems observed by study group

Figure 1: Funnel plot (P-chart) of percentage of patients seen by GP who were investigated by Full Blood Count (FBC)

Figure 2: Funnel plot (P-chart) of percentage of patients seen by GP who were investigated by Faecal Occult Blood test (FOBt)
