# Supplementary material for: Preliminary results of a feasibility study of the use of information technology for identification of suspected colorectal cancer in primary care: the CREDIBLE study
Source: Br J Cancer. 2015 Mar 3;112(Suppl 1):S70–6. doi: 10.1038/bjc.2015.45 (PMC4385979; doi:10.1038/bjc.2015.45)
Supplement: Supplementary Online Material [file bjc201545x4.doc]

Implementation process and examples of problems observed by study group

**Implementation process:**

1. Lead GP and practice manger are briefed on the study by the research team. A study pack, including Frequently Asked Questions and contact details of study team, is left at the practice, in hard copy and electronic copy.
2. The research team trains and supports a nurse to run a Colorectal Cancer Risk search at the GP practice using MSDI’s CRC module with built-in algorithm for NICE clinical factors and other risk factors.
3. An export list is generated and saved as an Excel version.
4. Nurse* checks each person flagged up in study age range (60-79) for investigations relating to flagged up clinical features and any other relevant clinical information which may help GP decide if patient may benefit from a check-up. Nurse notes whether patients appear to be eligible to be reviewed by GP and if not, why not.
5. Nurse prints a copy for the GP, and if possible meets GP to go through the list. If the GP is busy, the list is left with the practice manager and the practice is contacted again until the GP has been able to go through the first list. GPs are offered support to go through the first list.
6. Practice is contacted by the study team or nurse to see if GP has reviewed the list.
7. Once GP has reviewed the list and decided which patients to invite, research nurse or practice sends invitations and patient information sheet which have been agreed by Ethics Committee. Also included is an invitation to take part in an interview to discuss the process from a patient perspective.
8. Next month this process is repeated. Patient notes of newly flagged-up patients are reviewed.  Nurse checks status of previously flagged up patients and reminds GP if there are still patients to review.  Any patients who did not attend are sent a second invitation. Most patients are sent an appointment time, but this does vary by practice. Eg one practice asks patients to contact surgery to make an appointment and follows this up with a phone call as standard practice. In another practice with mostly ethnic minority patients, a receptionist speaking the appropriate language rings up patients who do not attend.
9. GP sees patients who are invited and, if appropriate, investigates further.
10. Nurse reviews and records outcomes, sending pseudonymised reports to the study team who monitor progress. GPs are supported, where appropriate, by the study team and the nurse. Study team liaises with GP practice if there are any concerns; nurse discusses individual patient cases with GP.

*In one practice, the GP, not a nurse, reviews the patients’ notes for eligibility.

**Examples of problems observed by study group**

1. **Software:**
2. Automated monthly electronic uploading of data from one clinical system to centrally held servers, followed by transference onto another clinical system server and then transference back to GP practice had to be smoothed out by the practices’ IT support unit. Initial teething problems meant that in some months the electronic records available to the study were not updated.
3. Several practices changed their clinical operating system: new procedures needed to be developed by the IT support unit, resulting in delays in starting or resuming searches.
4. Warehousing of data from SystmOne practices involved complex additional stages and safeguards to be developed before automated data extraction, processing and data downloading and running of searched.
5. **Problems for the practices:**

On first search we identified typically 50-60 patients for the GP to review. This presented some GPs with difficulties of:

1. Finding time to review the list. As the study progressed we sent monthly emails to remind practices of the number of patients flagged up this month and the number still waiting to be reviewed by the GP.
2. Potential clinical dilemma of what to do with patients flagged up – in many cases we identified patients meeting current NICE referral criteria who had not previously been considered by GP as appropriate for referral to rule out cancer.
3. Some practices struggled to find regular space and a spare computer for the nurse to carry out record reviews.
4. **Communication:**

The study worked best where we had good access to the GP involved and where the practice manager was supportive. When the lead GP was unable to review the patient list, the practice manager was often an ally in recruiting a substitute GP. However, in one case this occurred without the substitute GP being briefed about the study.

1. **Referrals:**

Individual cases highlighted that optimum referral pathways were not always followed. As the study progressed it became apparent that we needed to support some practices with a clear reminder of current NICE referral criteria and their rationale.
